# Human dimensions of wildlife conservation in Iran: Assessment of human-wildlife conflict in restoring a wide-ranging endangered species

**DOI:** 10.1371/journal.pone.0220702

**Published:** 2019-08-02

**Authors:** Saeideh Esmaeili, Mahmoud-Reza Hemami, Jacob R. Goheen

**Affiliations:** 1 Department of Zoology and Physiology, University of Wyoming, Laramie, United States of America; 2 Department of Natural Resources, Isfahan University of Technology, Isfahan, Iran; University of Tasmania, AUSTRALIA

## Abstract

Human-wildlife conflicts restrict conservation efforts, especially for wide-ranging animals whose home ranges overlap with human activities. We conducted a study to understand conflicts with, and factors influencing the perceived value of an expanding population of onagers (*Equus hemionus onager*) in local communities in southern Iran. We asked about locals’ perceptions of six potential management strategies intended to lessen human-onager conflict. We found that human-onager conflict was restricted to 45% of respondents within the Bahram-e-Goor Protected Area, all of whom were involved in farming or herding activities. Locals within the protected area were more knowledgeable about onagers and valued onagers more than those living outside the protected area. The perceived value of onagers increased with level of education, total annual income, and perceptions of onager population trends; the perceived value of onagers decreased with the magnitude of conflict between onagers and locals. To tolerate or avoid conflicts with onagers, locals were supportive of monetary compensation and changing from a traditional lifestyle to industrialized farming (for farmers) or livestock production (for herders) with the help of government; locals did not support selling land to the government. Our study is among the first in human-wildlife conflict and local attitudes towards an endangered species and its recovery in Iran. We conclude that current levels of human-onager conflict are relatively low and perceived value of onagers is still relatively high. Therefore, wildlife authorities should consider the development of mitigation strategies with local communities before conflicts intensify.

## Introduction

Increasingly, wide-ranging animals share landscapes with humans and their livestock, triggering human-wildlife conflict which can impart harm on both sides [1–3]. Humans may directly kill wildlife in retaliation for livestock or crop depredation, thereby suppressing wildlife populations [4,5]. Such retaliatory killings can lead to collapse of the species’ geographic range, restricting small and isolated populations to formally-protected areas [6,7]. Therefore, understanding and resolving human-wildlife conflict is a prerequisite for effective wildlife conservation in multi-use landscapes [8–10].

**Fig 1:**
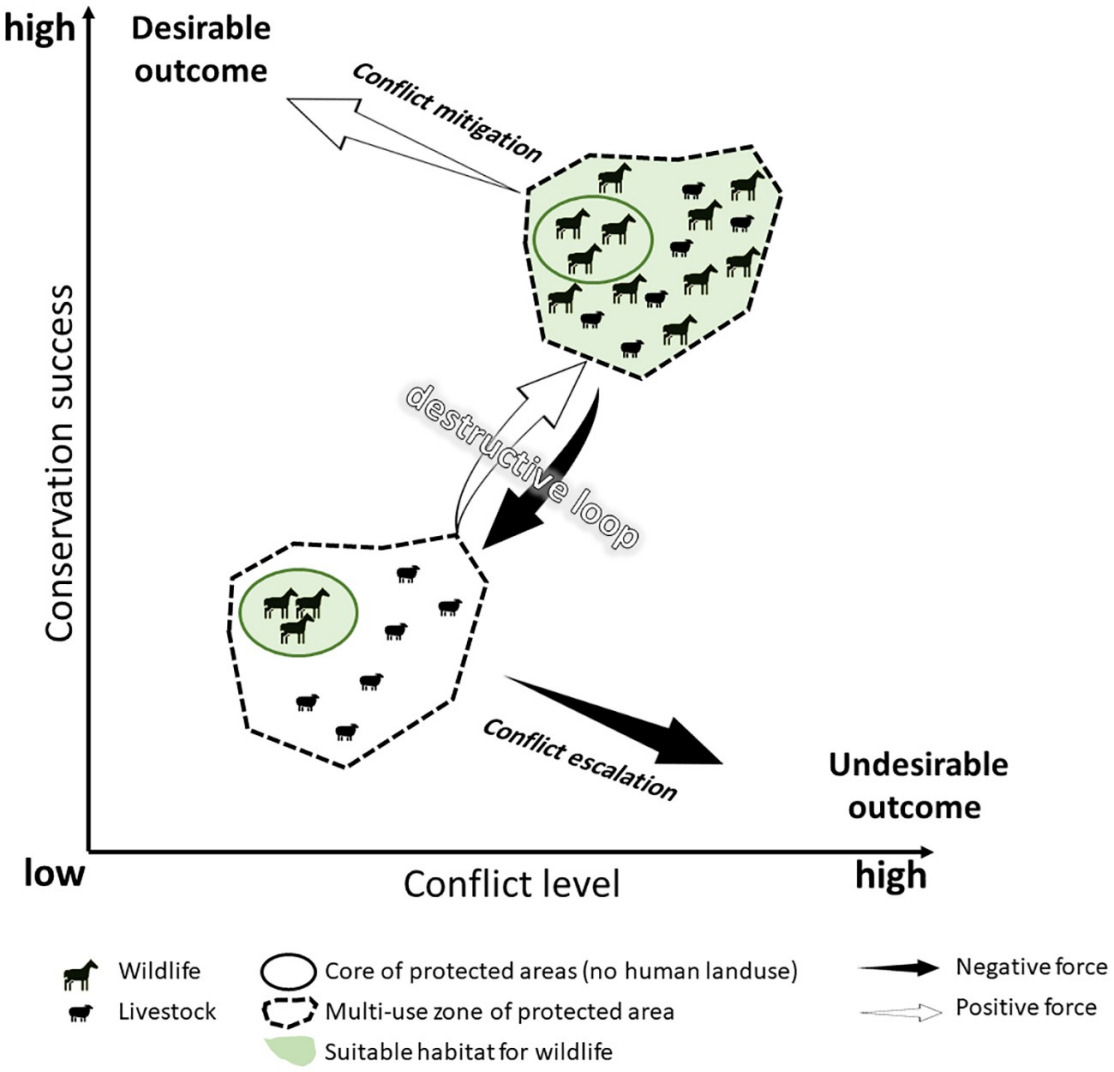
Possible outcomes of conservation activities for endangered species in multi-use landscapes.

Conservation efforts aimed at reducing human-wildlife conflict have been successful in bolstering population sizes and expanding geographic ranges for many of species [11]. Nevertheless, expansion of wildlife populations into multi-use landscapes can reignite conflicts, thereby creating a negative feedback between recovery of wildlife populations and human-wildlife conflicts. Ultimately, such negative feedbacks can inhibit conservation success [4,12](Fig 1). Because few protected areas are sufficiently large to conserve them [1,13,14], such destructive loops are a major challenge for wide-ranging species that depend on access to multi-use landscapes [15–17]. When stakeholders view human-wildlife conflict as a shared problem which all parties have a vested interest in solving, the destructive loops can be averted [5,16](Fig 1).

The black arrow shows a negative effect of conservation on human livelihoods, which can reduce or negate conservation efforts. A destructive loop is created when conservation success results in increased conflict levels, which results in a feedback of retaliatory killings and renewed conservation efforts. Although conflict escalation leads to conservation failure, conflict mitigation breaks the feedback loop and leads to conservation success.

Positive attitudes toward wildlife are a powerful driver for wildlife recovery [18]. The most viable approaches toward mitigating human-wildlife conflict are those in which negative outcomes for both humans and wildlife are reduced, or where common benefits make coexistence desirable (i.e., a “win-win outcome”, [5,19]). Anticipating conflicts can provide solutions before negative attitudes hinder dialogue. The first steps in managing conflict are to understand the nature of the conflict, local attitudes toward wildlife, and how wildlife impact the economic well-being of the local communities [20,21].

**Fig 2:**
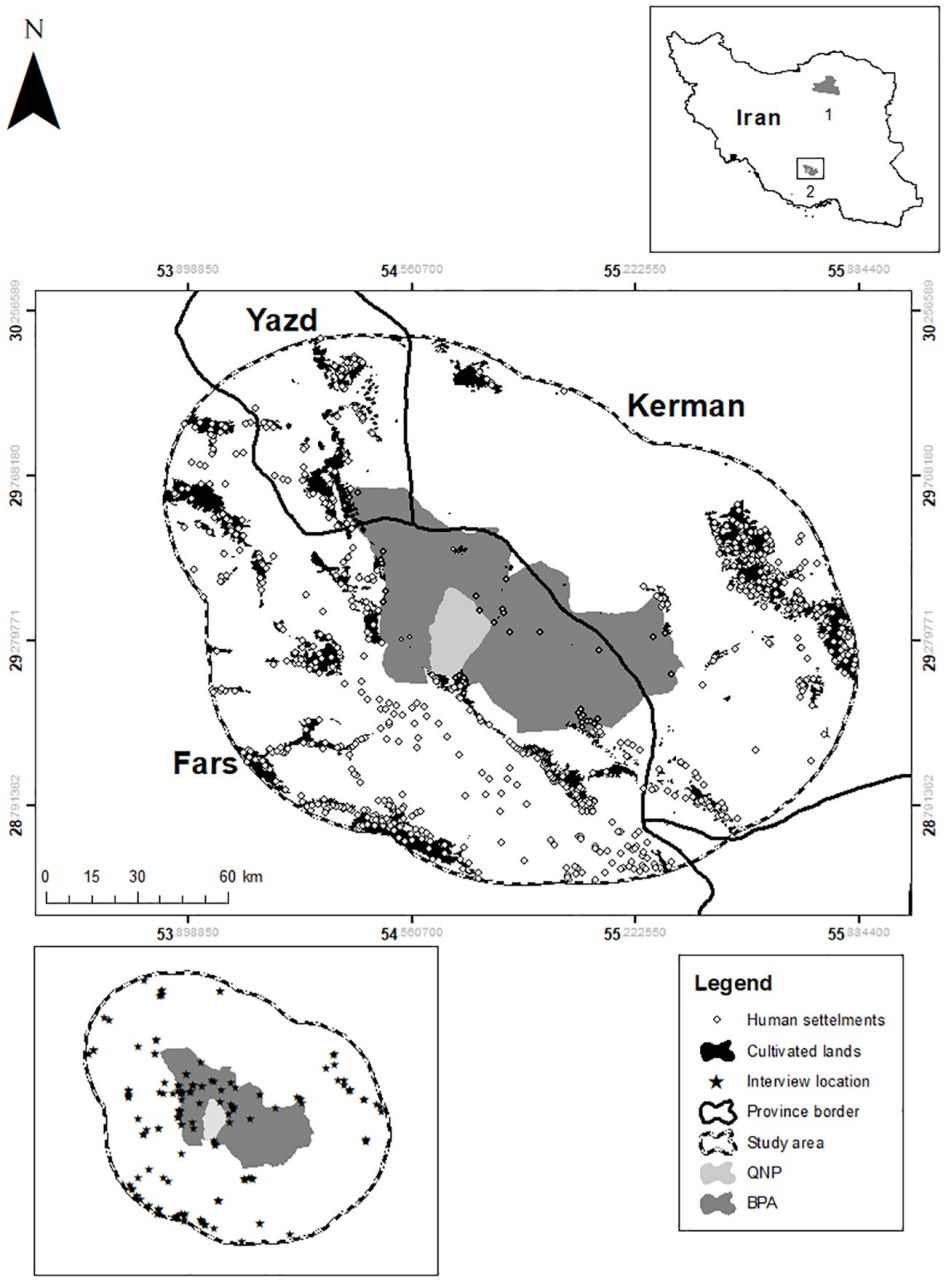
Current wild distribution of onager in Iran. (1) Touran Biosphere Reserve and (2) Bahram-e-Goor Protected Area (BPA) surrounding Qatrouiyeh National Park (QNP). Study area and location of interviews with local communities in BPA and a 50-kilometer surrounding buffer.

The Asiatic wild ass (*Equus hemionus*) is found throughout deserts and desert-steppes of Central Asia. With an estimated global population of 55,000, the species is still relatively abundant in some areas, but it occupies less than 3% of its historic range [22]. The Persian wild ass or onager (*E*. *h*. *onager*) is an endangered subspecies of Asiatic wild ass [22,23] that, over the last half century, declined precipitously because of poaching and reduced funds for conservation [24]. In 1997, a historically low population of 140 individuals was restricted to two protected area complexes: the Bahram-e-Goor Protected Area (BPA) and the Touran Biosphere Reserve in central Iran [25](Fig 2). In 1997, Qatrouiyeh National Park (in which livestock grazing is prohibited; QNP) was established within the BPA, which houses a growing onager population [26]. Onagers remain rare across the wider BPA, where livestock grazing is permitted.

The mechanisms of onager population suppression within BPA are not well understood [26], but poaching, agricultural expansion, and associated conflicts with pastoralists and their livestock are thought to have driven the decline [26]. Due to increasing human activity within BPA, onagers have become largely confined to QNP, where anti-poaching patrols are common. Further, QNP maintains artificial watering holes, and provides supplementary food in the dry season from May to December [26]. Consequently, onagers are now perceived as overabundant in QNP by wildlife managers (Pers. Comm. Iranian Department of Environment). Increasingly, however, farmers and nomadic herders (hereafter “herders”) in and around BPA are concerned about crop losses and competition between onagers and livestock, respectively (Esmaeili, personal observation). Thus, mitigating human-wildlife conflict should facilitate onager recovery within BPA as a first step to recovery in its historical range.

We sought to understand factors contributing to human-onager conflicts in rural communities in and around BPA. We used structured questionnaires to understand the nature and extent of the onager-human conflict, quantify local attitudes and knowledge, and assess support for potential management strategies. We hypothesized that (1) people residing within BPA would experience higher levels of conflict compared to those outside BPA; and (2) the occurrence and magnitude of conflicts would predict both people’s attitudes towards onagers, and their preferences for management strategies. Our study provides the first quantitative data on human-onager interactions to guide future recovery strategies for this endangered subspecies.

## Methods

Participants gave their consent verbally after being explained about the aim of the survey, topic of the different sections on the questionnaire, and how the interview would proceed. Consent was obtained by having the participants state that they agreed to participate. The data were collected and analyzed anonymously. The project, including the questionnaire and its consent acquisition procedure, received the approval from the Iranian Department of Environment (permission number: 33183/92 issued at 9 Dec 2013).

### Study area

We conducted our study in the Fars, Kerman, and Yazd Provinces on the central plateau of Iran. We were especially interested in differences between locals from the 3,747 km^2^ BPA and in a surrounding 50-kilometer buffer (26,938 km^2^; Fig 2). Bahram-e-Goor Protected Area has an arid climate (mean annual temperature 21 ± 1 °C, mean annual precipitation 186.11 ± 90.69 mm); September is the driest month and January is the wettest month (0.0 ± 0.0 mm and 38.13 ± 45.31 mm), respectively [27]. The area is characterized by desert steppe vegetation, dominated by *Artemisia sieberi*, *Zygophylllum eurypterum*, *Astragalus* spp., *Noea mucronate*, and sparse perennial trees such as mountain almond (*Amygdalus horrida*) and Turk terebinth pistache (*Pistacia atlantica*).

In 1972, BPA was established to protect onagers and other threatened species, including Indian gazelle (*Gazella bennettii*), wild sheep (*Ovis orientalis*), wild goat (*Capra aegagrus*), and Asian houbara bustard (*Chlamydotis maqueenii*). Additionally, BPA is home to wild boars (*Sus scrofa*) and gray wolves (*Canis lupus*), with the former regarded as a threat to crops [28] and the latter regarded as a threat to livestock [29] (there is no evidence of predation of wolves on onagers). Approximately 14 head of livestock (mainly sheep *Ovis aries* and goat *Capra hircus*, occasionally camel *Camelus dromedarius*) per square kilometer are permitted to graze inside the BPA (Fars Province Department of Environment, unpublished report). Frequent droughts are the main impediment to farming in the area, which have led to a transition from water-intensive crops such as wheat to drought-resistant orchards and livestock (Iranian Department of Environment, unpublished report).

Approximately 2,600 semi-nomadic pastoralists and small-scale farmers reside within BPA [30]. Outside BPA, about one-third of the total human population (ca. 470,000 individuals) lives in rural areas (centers of human activity with <15,000 inhabitants), whereas the other two-thirds live in urban centers with >15,000 inhabitants. Because the majority (60% of 190 individuals surveyed from urban centers) of urban residents had never heard of onagers, we focused on comparing responses from individuals residing within and along the boundary of BPA (hereafter “within BPA”) to those living outside BPA in rural areas (hereafter “outside BPA”).

### Sampling design

From January 2014 to July 2016, we conducted 102 interviews within BPA and 153 outside BPA. Only 2 (2%) respondents within BPA and 11 (7%) outside BPA were unaware of onagers, resulting in a sample size of 100 interviews from within BPA and 142 from outside BPA (S1 Table). We adopted a stratified random sampling design, in which the distribution of questionnaires was proportional to size of the rural population within the counties (which are nested within provinces) within our study area (Fig 2 and S1 Table). Outside BPA, and within each county, we randomly selected up to four villages in which to interview up to four people, each from a different household, without bias toward age or gender. We defined “households” as people from the same family and living within the same house. Within BPA, we randomly selected people across the entire area and along the boundary. Sampling intensity was 3.8% of the total population within BPA and 0.1% outside BPA (S1 Table).

Within BPA, we interviewed an additional 101 farmers and herders (16 farmers, 21 herders, and 64 individuals who were both farmers and herders) to quantify levels of agreement toward six strategies to reduce human-onager conflict.

### Questionnaire design

To ensure that our questionnaires addressed the objectives of our study, we conducted 25 pilot interviews with locals where we explored their views about their livelihoods, BPA, onagers, and other wildlife. We subsequently used that information to modify a pre-existing questionnaire used to assess public attitudes toward Asiatic wild ass in Mongolia [31]. To initiate interviews, we first asked if locals were aware of the existence of onagers. If so, we proceeded with our questionnaire (S1 Text); if not, we terminated the interview. Our questionnaire consisted of four sections to quantify information on (1) locals’ socio-economic background; (2) conflict with onagers; (3) knowledge about onagers; and (4) perceived value of onagers, plus an additional section (5) for the 101 additional respondents within BPA about acceptability toward the six potential strategies to reduce human-onager conflict (S1 Text).

We used responses from sections #1–3 as predictors for answers to questions in section #4 (on the perceived value of onagers, for 100 respondents within BPA and 142 respondents outside BPA), and in section #5 (acceptability of potential management strategies to reduce human-onager conflict for 101 respondents within BPA).

#### Section #1: Locals’ socio-economic background

We hypothesized that socio-economic background of locals would influence both the perceived value of onagers and acceptability of potential management strategies. We asked locals about their gender, age, and level of education (illiterate, primary and high school, university). We recorded livestock ownership (the number of livestock) for herders and farm size for farmers. We asked locals to categorize their total annual income in two categories of <$5000USD and >$5000 USD.

#### Section #2: Conflict with onagers

We hypothesized that conflict with onagers would influence both the perceived value of onagers and acceptability of potential management strategies. To assess the relative magnitude of human-onager conflict, we asked locals to rate the degree of conflict with onagers from no conflict to severe conflict coded from one to five. We asked locals about the occurrence, type, and timing of conflicts with onagers.

#### Section #3: Knowledge about onagers

We hypothesized that knowledge of locals would influence the perceived value of onagers. To quantify local knowledge about onagers, we asked if four incorrect statements about onager biology were true or false. When respondents stated that incorrect statements were correct, we recorded answers as a “0”. When respondents stated that incorrect statements were incorrect, we recorded answers as a “1”. We averaged these four answers to generate a knowledge score about onagers ranging from zero to one, indicating low and high levels of knowledge, respectively. Additionally, we asked about perceived population trends of onagers (in four categories of decreasing, increasing, stable, and do not know); we predicted that those who thought onager population had been decreasing would value the species more.

#### Section #4: Perceived value of onagers

To quantify the perceived value of onagers, we asked locals to rank agreement with fifteen statements from “strongly disagree” (coded as 1) to “strongly agree” (coded as 5). We averaged answers to these fifteen statements to produce a semi-continuous “value score” ranging from one to five (S2 Table, S1 Fig). We measured the internal consistency of the fifteen statements comprising the value score using Cronbach’s alpha consistency analysis (alpha function in psych Package in R) [32,33].

#### Section #5: Acceptability of potential management strategies

We inquired about the acceptability of six strategies aimed at reducing human-onager conflict, targeting only individuals within BPA (because individuals outside BPA did not experience conflict within onagers). We deliberately excluded fencing because we were most interested in management strategies that would not further fragment the distribution of onagers. The strategies were: (1) selling land to the government; (2) exchanging 50% farmland/pastureland within BPA for an equivalent amount of land outside BPA; (3) changing from a traditional farming/herding lifestyle to industrialized farming (for farmers) or livestock production (for herders) with the help of government; (4) accepting monetary compensation to tolerate onager conflict; (5) accepting a sedentary lifestyle instead of a nomadic one; and (6) supplementary feeding of livestock for a period in the year with the help of the government. The last two strategies were asked only from herders (who are nomadic within BPA) and not farmers (who are sedentary within BPA). We recorded responses in three categories: disagree, neutral, and agree. Unlike previous questions, we restricted responses to three categories because 5 categories were challenging for locals to understand.

### Statistical analyses

We used R software (versions 3.2.2 and 3.4.1, R Development Core Team 2013) for statistical analysis. To compare responses within and outside BPA, we used Chi-square (χ^2^) and Wilcoxon signed-ranks tests.

Using beta regression (Betareg package in R [34]), we related region (whether respondents were within or outside of BPA), socio-economic predictors (gender, age, level of education, total annual income), level of conflict with onagers, knowledge score, and perceived population trends of onagers to the value score. In calculating the value score, 36% of respondents were neither farmers nor herders, so we did not include livestock ownership or farm size in this regression model to include all the respondents in a single model. We standardized the semi-continuous value score between zero and one as a response variable in the beta regression. Beta regression is suitable for modeling heteroskedastic and non-normal data restricted between zero and one [35]. From generalized variance inflation factors, we detected no multicollinearity between predictors [36].

To predict factors influencing acceptability of management strategies, we related socio-economic predictors (age, total annual income, livestock ownership, and farm size) and level of conflict with onagers to three response categories of disagree, neutral, and agree for each strategy (as an ordinal response variable) using ordinal logistic regression models (lrm function in package rms [37]). Due to sample size constraints, we did not include level of education or gender in these regression models. We used likelihood ratio χ^2^ tests to select models and used R^2^ and a concordance-index (c-index) as a measure of predictive performance [37].

## Results

Human-onager conflict was restricted to respondents involved in farming or herding within BPA (S3 Table). No conflict with onagers was reported from outside BPA, whereas 45% of locals within BPA reported conflict with onagers (S2 Text).

Forty-five farmers and herders within BPA experienced conflict with onagers, categorizing it as little (31%), some (20%), high (20%), or severe (29%). Conflicts with onagers were largely a reoccurring issue spanning multiple years (median = 7 years, range 1 to 30 years). The proportion of all human-wildlife conflict attributed to onagers ranged between 1% and 100% (median = 30%). Most conflict with onagers were with respect to crop depredation, and occurred mostly in summer (58%), least in winter (20%), and at intermediate frequency in fall (40%) and spring (30%); 4% of respondents claiming that conflicts with onagers occurred year-round. The crops most susceptible to depredation were alfalfa (84%), wheat and barley (44%), fruit orchards and vegetables (22%), and corn (9%). Only three respondents (7%) reported conflicts over pasture.

Knowledge about onagers was poor overall. Locals within BPA were more knowledgeable (two questions were answered correctly, on average) and had higher knowledge scores (mean ± SD = 0.47 ± 0.32; Wilcoxon signed-ranks test, p < 0.001; S3 Text) than those outside (who answered one question correctly, on average; Table 1; mean ± SD = 0.29 ± 0.28).

**Table 1:** Four false statements comprising the “knowledge score” about onagers for respondents within and outside Bahram-e-Goor Protected Area (BPA) in southern Iran. N = 243 respondents. Chi-square tests compare results within and outside BPA.

Statement	Within BPA	Outside BPA	n	Χ^2^ (*p*)
False statement	*% answers correctly identifying statement as false*			
Onagers can run more than 100 km/hour.	35.45	24.66	243	3.57 (0.06)
Onager mares often give birth to 2 foals.	52.72	25.33	243	20.45 (<0.001)
Onagers need to drink only once a week.	61.82	24.67	243	36.38 (<0.001)
Onagers live in many areas of Iran.	40.91	45.33	243	0.50 (0.47)

Fifteen statements produced a consistent value score (standardized alpha value = 0.79, n = 249, average inter-item correlation = 0.21; S2 Table) ranging between 1.93 and 5.00. Value scores averaged 3.80 (SD = 0.45) and were slightly higher within BPA (mean ± SD: 3.86 ± 0.39; n = 100), than outside BPA (mean ± SD: 3.76 ± 0.48; n = 142; Wilcoxon signed-ranks test w = 8747, *p* = 0.05). Locals’ level of education, total annual income, and perceived onager population trends affected perceived value of onagers positively. Level of conflict with onagers negatively influenced the perceived value of onager, although explanatory power of the model was low (pseudo R^2^ = 0.21, z = 10.96, *p* < 0.001; Table 2).

**Table 2:** Parameter estimates of factors influencing perceived value of onagers resulted from a beta regression model (pseudo R^2^ = 0.21, z = 10.96, p<0.001) for locals within and outside the Bahram-e-Goor Protected Area, Iran. Positive estimates indicate positive association between a factor and perceived value of onagers.

Factor	*Estimate ± SE*	*Z*	*p*
intercept	-0.50±0.20	-2.53	0.01
outside BPA^1^	-0.08±0.14	-0.61	0.54
gender (male)^2^	0.04±0.12	0.35	0.72
age	0.04±0.06	0.66	0.51
education (primary and high school)^3^	0.22±0.15	1.30	0.19
education (university)^3^	0.65±0.22	3.00	**0.002**
total annual income (>$5000 USD)^4^	0.32±0.13	2.50	**0.01**
knowledge score	0.08±0.06	1.39	0.16
perceived onager population trend (decreasing)^5^	0.58±0.13	4.42	**<0.001**
perceived onager population trend (increasing)^5^	0.48±0.16	2.92	**0.003**
perceived onager population trend (stable)^5^	0.46±0.33	1.40	0.16
level of conflict with onagers	-0.17±0.06	-2.82	**0.004**

^1^ reference level: within BPA

^2^ reference level: female

^3^ reference level: illiterate

^4^ reference level: total annual income < $5000 USD

^5^ reference level: do not know

Presented with six potential management strategies, farmers and herders within BPA agreed most with accepting monetary compensation to tolerate onager conflicts and with changing from a traditional farming/herding lifestyle to industrialized farming (for farmers) or livestock production (for herders) with the help of government. Farmers and herders agreed least with selling land to the government. Farmers and herders were ambivalent towards exchanging 50% farmland/pastureland within BPA for land outside BPA and herders towards accepting a sedentary lifestyle instead of a nomadic one or supplementary feeding of livestock for a period in the year with the help of the government (Table 3).

**Table 3:** Acceptability of potential management strategies aimed at reducing human-onager conflict according to 101 farmers and herders within Bahram-e-Goor Protected Area (BPA), Iran.

Conservation strategy	Number of responses		
	*agree*	*neutral*	*disagree*
selling land to the government	4	6	91
exchanging 50% farmland/pastureland within BPA for an equivalent amount of land outside BPA	23	6	72
changing from a traditional farming/herding lifestyle to industrialized farming (for farmers) or livestock production (for herders) with the help of government	67	10	24
accepting monetary compensation to tolerate onager conflict	76	9	16
accepting a sedentary lifestyle instead of a nomadic one *	41	13	31
supplementary feeding of livestock for a period in the year with the help of the government *	50	6	29

*Only asked from herders.

Locals reporting high levels of conflict with onagers were less likely to agree with exchanging 50% of farmland/pastureland within BPA for land outside BPA, and were more likely to agree with monetary compensation to tolerate conflicts with onagers (Table 4). Age, livestock ownership, farm size, and total annual income were significant predictors of local agreement toward four of the six potential management strategies (Table 4). None of the selected predictors significantly affected locals’ agreement toward changing from a traditional farming/herding lifestyle to industrialized farming (for farmers) or livestock production (for herders) with the help of government, which was the second most popular strategy (Table 4).

**Table 4:** Parameter estimates (β±SE) and p-values (p) results of ordinal regression models to predict effects of socio-economic variables and level of conflict with onagers on accepting potential management strategies aimed at reducing human-onager conflict within Bahram-e-Goor Protected Area (BPA), Iran. Strategies included: (1) selling land to the government; (2) exchanging 50% farmland/pastureland within BPA for an equivalent amount of land outside BPA; (3) changing from a traditional farming/herding lifestyle to industrialized farming (for farmers) or livestock production (for herders) with the help of government;(4) accepting monetary compensation to tolerate onager conflict; (5) accepting a sedentary lifestyle instead of a nomadic one; and (6) supplementary feeding of livestock for a period in the year with the help of the government.

	*selling land*	*exchanging 50% of lands*	*changing from traditional farming/herding*	*accepting monetary compensation*	*accepting sedentary lifestyle*	*supplementary feeding of livestock*
intercept 1	-7.65±2.91 (0.008)					
intercept 2	-9.21±3.09 (0.003)					-2.31±1.19 (0.05)
age						0.03±0.02 (0.05)
livestock ownership	0.02±0.01 (0.004)				0.01±0.01 (0.04)	
farm size		0.09±0.04 (0.03)			*	*
total annual income (>$5000USD)	2.47±1.18 (0.035)					
level of conflict with onagers		-0.57±0.28 (0.04)		0.74±0.29 (0.01)		
model Likelihood ratio test χ^2^ (*p*)	20.67 (0.002)	14.48 (0.02)	2.95 (0.81)	14.43(0.02)	11.66(0.04)	9.71(0.08)
R^2^	0.41	0.21	0.041	0.19	0.16	0.14
c-index	0.88	0.71	0.58	0.73	0.70	0.67

* Predictors were not included in the analyses as the questions were asked from herders.

## Discussion

Our study is among the first to assess attitudes of local people toward human-wildlife conflict [38] and recovery of an endangered species in Iran. In Iran, wildlife conservation typically is conducted without involvement of local people [39]. Only recently has public awareness been raised from efforts to protect and recover highly charismatic, rare predators, namely Asiatic cheetah (*Acinonyx jubatus venaticus*) and Persian leopard (*Panthera pardus saxicolor*) [38,40]. However, focus on non-predatory species has been unusual thus far, despite their prominence in history, culture, and traditions [41]. Although recent progress has been made toward recovery of onagers within QNP, expansion into their former geographic range within Iran has been slow. We believe that a better understanding of human-onager relationships within local communities in and around BPA represents a starting point from which to further conservation and recovery efforts [42,43].

Although conflicts were restricted exclusively to rural respondents within BPA, these individuals did not value onagers less than respondents outside BPA. This lack of difference suggests that human-onager conflict is either low, or that even severe and reoccurring conflict with onagers has not yet lead to overly-negative attitudes [44]. Additionally, current levels of conflict with onagers did not drive agreement with potential management strategies. Not surprisingly, accepting monetary compensation to tolerate onager conflict was the most popular management strategy. Monetary compensation through direct payment is a common method to attenuate economic hardship caused by endangered wildlife [45–47]. In Iran, there is currently no systematic compensation scheme for wildlife, and monetary compensation by the Department of Environment in BPA has been restricted to a few special cases. Systematic compensation schemes require significant budgets, provide little incentive to invest into mitigation measures [48,49], and hence are not a sustainable option when aiming for endangered species recovery by a budget-starved agency. Financial compensation [47] to tolerate crop depredation from onagers could theoretically facilitate human-onager coexistence, but any financial compensation requires either local or international sources of revenue. Currently, Bahram-e-Goor and the Iranian Department of Environment lack sufficient funds to support and maintain such programs. Additionally, similar challenges exist for other wild population of onagers (in Touran Biosphere Reserve) and other wildlife species in Iran, which limits and complicates the revenue disbursement. We encourage the Iranian Department of Environment create compensation programs either through international funding (agencies, donations) or by promoting sustainable recreational activities in the protected areas, but we cannot rely on compensation programs to solve human-onager conflicts in the short-term.

Farmers and herders seem willing to switch from a traditional farming/herding lifestyle to a more industrialized form of crop and livestock production. If managed well, this could reduce the need for land conversion and minimize competition for pasture between wild and domestic ungulates. One potential drawback of industrialized farming might be increased human presence and transportation around onager habitats. However, the effects of such changes need to be carefully evaluated and local people and conservationists must come to a common understanding of the socio-economic, cultural, and ecological consequences of such changes. Selling or exchanging land from inside BPA for land outside BPA, on the other hand, received little support. Although separating wildlife and people can be highly effective in reducing conflict, forcing people to leave can escalate human-wildlife conflict [50,51].

Both of the most supported strategies (i.e. accepting monetary compensation to tolerate onager conflict and changing from a traditional farming/herding lifestyle to industrialized farming (for farmers) or livestock production (for herders) with the help of government) entail financial incentives. Critically, locals were less supportive of accepting a sedentary lifestyle instead of a nomadic one when financial incentives were not involved. So, satisfying the economic needs (or desires) of local communities may assist in reaching a common solution in human-wildlife conflicts [5,47].

Given the localized nature of human-onager conflict, monetary compensation through insurance schemes may be the most cost-efficient means of conflict reduction. Insurance schemes do not require government support necessarily. However, insurance schemes additionally require that locals protect their farms to be eligible for coverage offered by insurance (Esmaeili, personal communication). Advice on fence design and affordable technologies, like solar-powered electric fencing, could help reduce conflicts by both onagers and wild boars (which are widespread and consume crops throughout Iran [28] and the study area; Esmaeili, personal observation). However, fencing should be employed only as a last resort at small spatial scales around farms to protect crops. Fences should not be constructed around protected areas, as it would result in further fragmentation of the onager’s geographic distribution [52].

Although they were aware of onagers, knowledge scores of rural respondents both within and outside BPA were low; notably, onagers were viewed as widely distributed throughout Iran (Table 1). Our study highlighted that there is very little awareness for the precarious conservation status of onagers, as the majority of urban residents were unaware of the species’ existence. Such low awareness likely reflects the restricted distribution of onagers [25]. The remoteness of BPA and the small numbers of onagers within it provided little opportunity for direct interaction, the key source of respondents’ knowledge about onagers (S3 Text). Complicating matters further, in Farsi the word for onager and zebra is the same, creating confusion about the basic identity of the species. While Iranians distinguish between donkeys and domestic horses, there is a single word for all wild species of Family Equidae: “goor-e-khar”. Given the low awareness about onagers among urban residents in the periphery of BPA, one can assume that there is almost no awareness about this species among the remaining, largely urban population in Iran (73%, [40]). Therefore, and currently, little public support can be expected from the civil society [53]. The future of onager conservation may thus partially rely on the international conservation community, at least in the immediate future.

Currently, human-onager conflict is restricted within BPA; presently, there is little evidence for conflict escalation over onager conservation. As onager populations increase and expand outside QNP, however, these problems can be expected to intensify. To avoid entering a destructive loop (Fig 1), national and local authorities need to develop and support mitigation strategies together with local communities. The first step toward implementing mitigation strategies would be identifying hotspots of conflict and helping locals protect their lands to initiate compensation through insurance schemes. The Iranian Department of Environment would call for volunteers to transition from traditional farming or herding lifestyles to industrialized crop and livestock production after performing environmental impact assessments. Short-term and long-term action plans with attainable and measurable outcomes should be developed to implement the mitigation strategies. This should done while the conflict is still low and support for onager conservation is still relatively high.

## Supporting information

S1 Table The total rural population of the study area residing within and along the boundary of Bahram-e-Goor Protected Area (within BPA) and outside BPA. A Stratified random sampling approach based on population size of counties was used to select the number of interviewees at each category. N questionnaires represent the total number of interviews (regardless of respondents’ familiarity with onagers).
(DOCX)

S2 Table Internal consistency of the fifteen statements comprising value score, reflecting perceived value of onagers in and outside Bahram-e-Goor Protected Area, Iran. Cronbach’s alpha consistency analysis is used to generate Cronbach’s alpha score, which indicates high consistency between components of the value score.
(DOCX)

S3 Table Locals’ socio-economic background of respondents to the questionnaires in and around Bahram-e-Goor Protected Area, Iran. N questionnaires represent the total number of interviews (regardless of respondents’ familiarity with onagers).
(DOCX)

S1 Fig Response of locals to fifteen statements comprising value score, reflecting perceived value of onagers within and outside Bahram-e-Goor Protected Area, Iran. We asked locals to rank agreement with fifteen statements from “strongly disagree” (coded as 1) to “strongly agree” (coded as 5).
(DOCX)

S1 Text Questionnaire for proposed human-onager survey in Bahram-e-Goor Protected Area. (Farsi version of the questionnaire was used in interviews).
(DOCX)

S2 Text Human-onager conflict patterns in Bahram-e-Goor Protected Area, Iran. (DOCX)

S3 Text Locals’ knowledge about onagers in and around Bahram-e-Goor Protected Area, Iran. (DOCX)
