# Supplementary material for: Human dimensions of wildlife conservation in Iran: Assessment of human-wildlife conflict in restoring a wide-ranging endangered species
Source: PLoS One. 2019 Aug 2;14(8):e0220702. doi: 10.1371/journal.pone.0220702 (PMC6677293; doi:10.1371/journal.pone.0220702)
Supplement: S1 Table — A Stratified random sampling approach based on population size of counties was used to select the number of interviewees at each category. N questionnaires represent the total number of interviews (regardless of respondents’ familiarity with onagers). (DOCX) [file pone.0220702.s001.docx]

**S1 Table. The total rural population of the study area residing within and along the boundary of Bahram-e-Goor Protected Area (within BPA) and outside BPA.** A Stratified random sampling approach based on population size of counties was used to select the number of interviewees at each category. N questionnaires represent the total number of interviews (regardless of respondents’ familiarity with onagers).

| **County (Dehestan)** | **District (Sharestan)** | **Province** | **Inhabitants in 2006** | **N Questionnaires** | **% of total population interviewed** |
| --- | --- | --- | --- | --- | --- |
| ***Within BPA*** | | | | | |
| Villages nomads | NA | NA | 2,671 | 102 | **3.8** |
| ***Outside BPA*** | | | | | |
| Tojerdi | Bavanat | Fars | 6,347 | 4 | 0.06 |
| Bakhtajerd | Darab | Fars | 8,145 | 5 | 0.06 |
| Balesh | Darab | Fars | 8,016 | 4 | 0.05 |
| Fasaroud | Darab | Fars | 2,379 | 2 | 0.08 |
| Hoshivar | Darab | Fars | 9,119 | 6 | 0.07 |
| Kouhestan | Darab | Fars | 1,563 | 1 | 0.06 |
| Paskhan | Darab | Fars | 10,386 | 7 | 0.07 |
| Qalebiaban | Darab | Fars | 3,168 | 2 | 0.06 |
| Qariekheir | Darab | Fars | 6,586 | 4 | 0.06 |
| Ij | Estahban | Fars | 3,661 | 8 | 0.22 |
| Kheir | Estahban | Fars | 1,268 | 0 | 0.00 |
| Roniz | Estahban | Fars | 13 | 0 | 0.00 |
| Qarebolaq | Fasa | Fars | 17,396 | 13 | 0.07 |
| Sheshdeh | Fasa | Fars | 208 | 0 | 0.00 |
| Abadetashk | Neiriz | Fars | 4 | 0 | 0.00 |
| Bakhtegan | Neiriz | Fars | 7,082 | 6 | 0.08 |
| Dehchah | Neiriz | Fars | 2,748 | 2 | 0.07 |
| Hannad | Neiriz | Fars | 9,594 | 7 | 0.07 |
| Horagan | Neiriz | Fars | 1,110 | 1 | 0.09 |
| Moshgan | Neiriz | Fars | 167 | 4 | 2.40 |
| Qatrouyeh | Neiriz | Fars | 3,685 | 3 | 0.08 |
| Rostaq | Darab | Fars | 2,506 | 3 | 0.12 |
| Rostaq | Neiriz | Fars | 5,511 | 6 | 0.12 |
| Rizab | Neiriz | Fars | 10,832 | 7 | 0.06 |
| Estabraq | Shahr-e-Babak | Kerman | 9 | 0 | 0.00 |
| Khorsand | Shahr-e-Babak | Kerman | 931 | 4 | 0.43 |
| Golestan | Sirjan | Kerman | 2,638 | 3 | 0.11 |
| Mahmoudabad | Sirjan | Kerman | 7,982 | 4 | 0.05 |
| Malekabad | Sirjan | Kerman | 7,656 | 5 | 0.06 |
| Najafabad | Sirjan | Kerman | 4,980 | 11 | 0.22 |
| Sharifabad | Sirjan | Kerman | 8,758 | 6 | 0.07 |
| Zeidabad | Sirjan | Kerman | 3,021 | 7 | 0.23 |
| Chahak | Khatam | Yazd | 5,225 | 5 | 0.10 |
| Fathabad | Khatam | Yazd | 3,035 | 10 | 0.33 |
| Harabarjan | Khatam | Yazd | 0 | 0 | 0.00 |
| Isar | Khatam | Yazd | 0 | 0 | 0.00 |
| **Total** |  |  | **165,729** | **153** | **0.10** |
