# Supplementary material for: Human dimensions of wildlife conservation in Iran: Assessment of human-wildlife conflict in restoring a wide-ranging endangered species
Source: PLoS One. 2019 Aug 2;14(8):e0220702. doi: 10.1371/journal.pone.0220702 (PMC6677293; doi:10.1371/journal.pone.0220702)
Supplement: S2 Table — Cronbach’s alpha consistency analysis is used to generate Cronbach’s alpha score, which indicates high consistency between components of the value score. (DOCX) [file pone.0220702.s002.docx]

**S2 Table- Internal consistency of the fifteen statements comprising value score, reflecting perceived value of onagers within and outside Bahram-e-Goor Protected Area, Iran.** Cronbach’s alpha consistency analysis is used to generate Cronbach’s alpha score, which indicates high consistency between components of the value score.

| **Score / statements** | **Cronbach's Alpha if statement deleted** |
| --- | --- |
| ***value score (standardized Cronbach’s alpha = 0.80, n=249)*** | |
| The presence of onagers is important for Iran's nature. | 0.77 |
| It is important to maintain onagers for our children. | 0.76 |
| It is not necessary to protect onagers in Iran, because large populations are elsewhere.* | 0.78 |
| Onagers are beautiful animals. | 0.77 |
| I am proud that Iran has onagers. | 0.76 |
| Onagers only cause problems for us.* | 0.78 |
| I like onagers, but not near my home.* | 0.79 |
| Onagers should be fully protected in Bahram-e-Goor. | 0.79 |
| These many onagers should not be in Bahram-e-Goor.* | 0.80 |
| Local people should be allowed to hunt onagers.* | 0.79 |
| More money should be spent on conservation/protection of onagers. | 0.79 |
| Local people are willing to help protect onagers. | 0.79 |
| Onagers should be restricted to the Qatrouyeh National Park.* | 0.80 |
| Poaching of onagers needs to be counteracted by better enforcement. | 0.79 |
| It is important to protect some areas like Qatrouyeh National Park primarily for onagers. | 0.79 |

*Coding reversed
