# Supplementary material for: Human dimensions of wildlife conservation in Iran: Assessment of human-wildlife conflict in restoring a wide-ranging endangered species
Source: PLoS One. 2019 Aug 2;14(8):e0220702. doi: 10.1371/journal.pone.0220702 (PMC6677293; doi:10.1371/journal.pone.0220702)
Supplement: S3 Table — N questionnaires represent the total number of interviews (regardless of respondents’ familiarity with onagers). (DOCX) [file pone.0220702.s003.docx]

**S3 Table. Locals’ socio-economic background of respondents to the questionnaires in and around Bahram-e-Goor Protected Area (BPA), Iran.** N questionnaires represent the total number of interviews (regardless of respondents’ familiarity with onagers).

| **Variables** | **Within BPA** | **Outside BPA** | **Total** |
| --- | --- | --- | --- |
| **N interviews** | 102 | 153 | 255 |
| **Gender composition (%)** |  |  |  |
| Males | 77.45 | 71.89 | 74.12 |
| Females | 22.55 | 28.10 | 25.88 |
|  |  |  |  |
| **Age** |  |  |  |
| Mean | 44 | 39.48 | 41.28 |
| SD | 14.97 | 14.75 | 14.97 |
| Min | 16 | 20 | 16 |
| Max | 88 | 85 | 88 |
|  |  |  |  |
| **Education (%)** |  |  |  |
| Illiterate | 15.69 | 15.03 | 15.29 |
| School | 72.55 | 69.28 | 70.59 |
| University | 11.76 | 15.69 | 14.12 |
|  |  |  |  |
| **Occupation (%)** |  |  |  |
| Farmer | 10.78 | 31.37 | 22.35 |
| Herder | 16.66 | 3.92 | 9.02 |
| Farmer and Herder | 94.11 | 8.50 | 32.16 |
| Government | 0 | 5.23 | 3.14 |
| Housewife | 0.98 | 20.26 | 12.55 |
| Other | 3.92 | 32.03 | 20.78 |
|  |  |  |  |
| **Farm size**^1^ |  |  |  |
| Mean | 6.68 | 4.10 | 5.74 |
| SD | 12.44 | 5.08 | 10.17 |
| Min | 0.50 | 1 | 0.50 |
| Max | 80 | 35 | 80 |
| N | 70 | 48 | 118 |
|  |  |  |  |
| **Livestock ownership**^2^ |  |  |  |
| Mean | 101.4 | 54.23 | 86.2 |
| SD | 99.87 | 82.40 | 86.93 |
| Min | 3 | 1 | 1 |
| Max | 700 | 500 | 700 |
| N | 88 | 39 | 128 |

1 cultivation type (n=153): wheat (53%), barley (39%), pistachio (39%), alfalfa (25%), other fruit trees (25%), corn (23%), pomegranate (20%), others (8%).

^2^ Livestock type (n=128): herds of sheep and goat (55%), sheep (34%), goat (5%), camel (1%), others (5%).
