# Supplementary material for: Human dimensions of wildlife conservation in Iran: Assessment of human-wildlife conflict in restoring a wide-ranging endangered species
Source: PLoS One. 2019 Aug 2;14(8):e0220702. doi: 10.1371/journal.pone.0220702 (PMC6677293; doi:10.1371/journal.pone.0220702)
Supplement: S1 Fig — We asked locals to rank agreement with fifteen statements from “strongly disagree” (coded as 1) to “strongly agree” (coded as 5). (DOCX) [file pone.0220702.s004.docx]

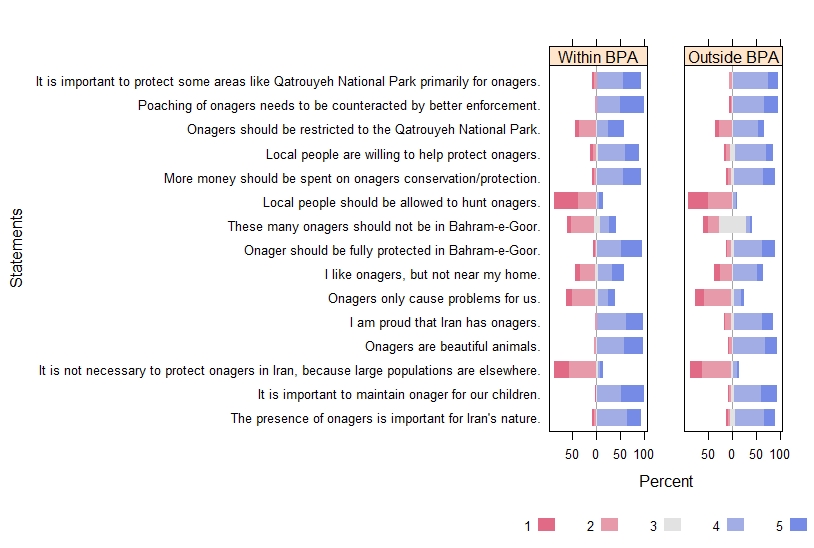
**S1 Fig. Response of locals to fifteen statements comprising value score, reflecting perceived value of onagers within and outside Bahram-e-Goor Protected Area (BPA), Iran.** We asked locals to rank agreement with fifteen statements from “strongly disagree” (coded as 1) to “strongly agree” (coded as 5).
