# Supplementary material for: Human dimensions of wildlife conservation in Iran: Assessment of human-wildlife conflict in restoring a wide-ranging endangered species
Source: PLoS One. 2019 Aug 2;14(8):e0220702. doi: 10.1371/journal.pone.0220702 (PMC6677293; doi:10.1371/journal.pone.0220702)
Supplement: S1 Text — (Farsi version of the questionnaire was used in interviews). (DOCX) [file pone.0220702.s005.docx]

**S1 Text. Questionnaire for proposed human-onager survey in Bahram-e-Goor Protected Area.**

(Farsi version of the questionnaire was used in interviews)

Village / GPS point: _______

Municipality: _____________

Date: _______

***Section #1: Locals’ socio-economic background***

Age: _____

Sex: male female

Education: _________

Main occupation:____________________________________________________________________

***If farmer,***

What does he / she cultivate: _______________________________________________________

Size of land:____________

How much is his /her annual harvest (tons/ hectare):____________________________________

Location of the farmland: ____________

***If herder;***

how many livestock: ______________________________________________________________

what type: _____________________________________________________________________

Answering the following question is optional:

- How much is your family income in a month/a year (we asked them in IRI Rials)

1. Less than $5000 in year
2. More than $5000 in year

***Section #2: Conflict with onagers***

1. Do you experience any conflicts with wildlife in last 10 years? yes no
2. How do you rank your level of conflicts with onagers?

| no conflict | little conflict | some conflict | high conflict | sever conflict |
| --- | --- | --- | --- | --- |
| 1 | 2 | 3 | 4 | 5 |

1. How many years have you been experiencing onager damage?
2. What percent of your overall wildlife damage is due to onager? __________
3. What is the main damage by onagers?

barley & wheat alfalfa corn vegetables fruit trees nut trees pastureland (competition with livestock) potatoes other:__________

1. What season do most onagers damages happen:

spring summer fall winter all year round

1. Are there more damages now than in the past?

Yes No

Why?________________________________________________________________________________________________________________________________________________________________________________________________________________________________________

10. What do you do to avoid damages by onagers?

_____________________________________________________________________________________________________________________________________________________________________________________________________________________________________________

12. What would you like DoE or the National park to do to reduce wildlife / onagers damages?

______________________________________________________________________________________________________________________________________________________________

13. Have you heard about any dead onagers during the last 5 years?

Yes No

Where?_______________________________________________________________________________________________________________________________________________________

***Section #3: Knowledge about onagers***

1. Which of the following statements do you consider correct?

| *Knowledge score* | Not correct | correct | do not know |
| --- | --- | --- | --- |
| 1- Onager can run more than100 km/hour. | 1 | 0 | 0 |
| 2- Onager mares often give birth to 2 foals. | 1 | 0 | 0 |
| 3- Onagers need to drink only once a week. | 1 | 0 | 0 |
| 4- Onagers live in many areas of Iran. | 1 | 0 | 0 |

1. Do you think onager numbers are?

increasing remain the same decreasing do not know

1. What do you think has the strongest influence on onager numbers in Bahram-o-Goor?
2. From where did you get your knowledge about onager?

own observation family school books newspaper radio TV DoE / rangers other:

***Section #4: Perceived value of onagers***

How do you feel about the following statements?

|  | strongly disagree | disagree | neutral | agree | strongly agree |
| --- | --- | --- | --- | --- | --- |
| 1- The presence of onagers is important for Iran's nature. | 1 | 2 | 3 | 4 | 5 |
| 2- It is important to maintain onager for our children. | 1 | 2 | 3 | 4 | 5 |
| 3- It is not necessary to protect onagers in Iran, because large populations are elsewhere. | 1 | 2 | 3 | 4 | 5 |
| 4- Onagers are beautiful animals. | 1 | 2 | 3 | 4 | 5 |
| 5- I am proud that Iran has onagers. | 1 | 2 | 3 | 4 | 5 |
| 6- Onagers only cause problems for us. | 1 | 2 | 3 | 4 | 5 |
| 7- I like onagers, but not near my home. | 1 | 2 | 3 | 4 | 5 |
| 8- Onager should be fully protected in Bahram-e-Goor. | 1 | 2 | 3 | 4 | 5 |
| 9- These many onagers should not be in Bahram-e-Goor. | 1 | 2 | 3 | 4 | 5 |
| 10- Local people should be allowed to hunt onagers | 1 | 2 | 3 | 4 | 5 |
| 11- More money should be spent on onagers conservation/protection of onagers. | 1 | 2 | 3 | 4 | 5 |
| 12- Local people are willing to help protect onagers. | 1 | 2 | 3 | 4 | 5 |
| 13- Onagers should be restricted to the Qatrouyeh National Park. | 1 | 2 | 3 | 4 | 5 |
| 14- Poaching of onagers needs to be counteracted by better enforcement. | 1 | 2 | 3 | 4 | 5 |
| 15- It is important to protect some areas like Qatrouiyeh National Park primarily for onagers. | 1 | 2 | 3 | 4 | 5 |

***Section #5: Acceptability of potential management strategies***

How are you willing to take the following decisions if government supports you?

|  | disagree | neutral | agree |
| --- | --- | --- | --- |
| 1- selling land to the government | 0 | 1 | 2 |
| 2- exchanging 50% farmland/pastureland within BPA for an equivalent amount of land outside BPA | 0 | 1 | 2 |
| 3- changing from a traditional farming/herding lifestyle to industrialized farming (for farmers) or livestock production (for herders) with the help of government | 0 | 1 | 2 |
| 4- accepting monetary compensation to tolerate onager damage | 0 | 1 | 2 |
| 5- accepting a sedentary lifestyle instead of a nomadic one | 0 | 1 | 2 |
| 6- supplementary feeding of livestock for a period in the year with the help of the government | 0 | 1 | 2 |
