# Supplementary material for: Human dimensions of wildlife conservation in Iran: Assessment of human-wildlife conflict in restoring a wide-ranging endangered species
Source: PLoS One. 2019 Aug 2;14(8):e0220702. doi: 10.1371/journal.pone.0220702 (PMC6677293; doi:10.1371/journal.pone.0220702)
Supplement: S2 Text — (DOCX) [file pone.0220702.s006.docx]

**S2 Text. Human-onager conflict patterns in Bahram-e-Goor Protected Area, Iran.**

Methods used by local farmers and herders to avert or reduce onager conflicts were (n = 40): filling complaints to the Iranian Department of Environment (27%), fencing the property (17%), digging deep trench around their farms (12%), making fire or noise, using scarecrow and standing watch (12%). However, a majority of 45% stated they did nothing to address the problem.

Expected solutions from the Iranian Department of Environment by the affected farmers were: keeping onagers away from people’s property by providing supplementary food and water (38%), fencing Qatrouiyeh National Park to avoid onagers conflicts (28 %), monetary compensation of the conflict (26%), helping people fence or dig trench around their properties (26%), recruiting more rangers to protect people’s property (11 %), and purchasing people’s property (4%).

Within the Bahram-e-Goor Protected Area 52% of respondents had known about onager kills in the past 5 years. The reasons of the death, as reported by 42 of the locals, were roadkill (43%), poaching (29%), both roadkill and poaching (17%), and other natural causes (12%). Outside the Bahram-e-Goor Protected Area, only 25% of locals heard about onager kills, of which 20 respondents knew about the reason of the death: roadkill (50%), poaching (35%), and other (15%).
