# Supplementary material for: Human dimensions of wildlife conservation in Iran: Assessment of human-wildlife conflict in restoring a wide-ranging endangered species
Source: PLoS One. 2019 Aug 2;14(8):e0220702. doi: 10.1371/journal.pone.0220702 (PMC6677293; doi:10.1371/journal.pone.0220702)
Supplement: S3 Text — (DOCX) [file pone.0220702.s007.docx]

**S3 Text. Locals’ knowledge about onagers in and around Bahram-e-Goor Protected Area, Iran.**

The main source of knowledge about onager for locals within BPA was own observations (92%), followed by family and friends (35%), DoE staff /rangers (27%), and TV (23%). Locals outside BPA knew about onager from TV (73%), direct observation (43%), family and friends (35%), DoE staff/rangers (11%), and books (11%). Other sources of knowledge were reported by less than 5% of respondents.

Onager population estimates of locals within BPA averaged 317.80 ± 203.24 (n = 86), and 54.0% reported (n = 100) an increasing population trends in recent years (decreasing: 30%, stable: 7%, do not know: 9%). Few people outside BPA gave any population estimate (267.50 ± 422.32, n = 25) and 89% of respondents (n = 142) were not aware of the increasing trend of the onager population (decreasing: 28%, stable: 1%, do not know: 61%).

Sixty four percent of respondents gave an answer to the open-ended question asking them about the strongest influence on onager numbers in BPA. We pooled all answers, resulting in the following list of positive and negative influences: protection (27%), change in people’s attitude (4%), poaching (22%), habitat lost due to anthropogenic factors (2%), droughts (18%), and natural effects (1%). However, 48% of respondents did not have any idea about this question.
